# Recent updates on different methods of pretreatment of lignocellulosic feedstocks: a review

**DOI:** 10.1186/s40643-017-0137-9

**Published:** 2017-01-18

**Authors:** Adepu Kiran Kumar, Shaishav Sharma

**Affiliations:** Bioconversion Technology Division, Sardar Patel Renewable Energy Research Institute, Vallabh Vidyanagar, Anand, 388 120 Gujarat India

**Keywords:** Pretreatment, Lignocellulosic biomass, Cellulose, Lignin, Reducing sugars

## Abstract

Lignocellulosic feedstock materials are the most abundant renewable bioresource material available on earth. It is primarily composed of cellulose, hemicellulose, and lignin, which are strongly associated with each other. Pretreatment processes are mainly involved in effective separation of these complex interlinked fractions and increase the accessibility of each individual component, thereby becoming an essential step in a broad range of applications particularly for biomass valorization. However, a major hurdle is the removal of sturdy and rugged lignin component which is highly resistant to solubilization and is also a major inhibitor for hydrolysis of cellulose and hemicellulose. Moreover, other factors such as lignin content, crystalline, and rigid nature of cellulose, production of post-pretreatment inhibitory products and size of feed stock particle limit the digestibility of lignocellulosic biomass. This has led to extensive research in the development of various pretreatment processes. The major pretreatment methods include physical, chemical, and biological approaches. The selection of pretreatment process depends exclusively on the application. As compared to the conventional single pretreatment process, integrated processes combining two or more pretreatment techniques is beneficial in reducing the number of process operational steps besides minimizing the production of undesirable inhibitors. However, an extensive research is still required for the development of new and more efficient pretreatment processes for lignocellulosic feedstocks yielding promising results.

## Background

Lignocellulosic feedstock represents an extraordinarily large amount of renewable bioresource available in surplus on earth and is a suitable raw material for vast number of applications for human sustainability. The main composition of lignocellulosic feedstocks is cellulose, hemicellulose, and lignin (Table 1). However, many obstacles are associated with effective utilization of lignocellulosic materials. Some of the major factors are the recalcitrance of the plant cell wall due to integral structural complexity of lignocellulosic fractions and strong hindrance from the inhibitors and byproducts that are generated during pretreatment. In addition, few more challenges still remain, like understanding the physicochemical architecture of feedstock cell walls, suitable pretreatment method and extent of cell wall deconstruction for generation of value-added products etc.

**Table 1:** Cellulose, hemicellulose, and lignin content in common lignocellulosic feedstocks

Lignocellulosic feedstocks	Cellulose (%)	Hemicellulose (%)	Lignin (%)
Sugar cane bagasse	42	25	20
Sweet sorghum	45	27	21
Hardwood	40–55	24–40	18–25
Softwood	45–50	25–35	25–35
Corn cobs	45	35	15
Corn stover	38	26	19
Rice Straw	32	24	18
Nut shells	25–30	25–30	30–40
Newspaper	40–55	25–40	18–30
Grasses	25–40	25–50	10–30
Wheat straw	29–35	26–32	16–21
Banana waste	13.2	14.8	14
Bagasse	54.87	16.52	23.33
Sponge gourd fibers	66.59	17.44	15.46
Agricultural residues	5–15	37–50	25–50
Hardwood	20–25	45–47	25–40
Softwood	30–60	40–45	25–29
Grasses	0	25–40	35–50
Waste papers from chemical pulps	6–10	50–70	12–20
Newspaper	12	40–55	25–40
Sorted refuse	60	20	20
Leaves	15–20	80–85	0
Cotton seed hairs	80–95	5–20	0
Paper	85–99	0	0–15
Switch grass	45	31.4	12

There are several criteria for the selection of a suitable pretreatment method: (a) the selected method should avoid the size reduction of biomass particles, (b) hemicellulose fraction must be preserved, (c) minimize the formation of degradation products, (d) minimize the energy demands and lastly, (e) should involve a low-cost pretreatment catalyst and/or inexpensive catalyst recycle and regeneration of high-value lignin co-product (Wyman 1999). The result of the pretreatment must not only defend but also justify its impact on the cost of downstream processing steps and the tradeoff between operating costs, capital costs, biomass costs, etc. (Lynd et al. 1996).

The pretreatment techniques for overcoming biomass recalcitrance are broadly divided into two classes: biochemical and thermochemical (Laser et al. 2009). Based on the operating temperatures, thermochemical pretreatment is again of two types: pyrolysis and gasification. The advantage of thermochemical conversion is that it is a fast process with low residence time and is able to handle a broad range of feedstock in a continuous manner, but major drawback is its non-specific nature of biomass deconstruction. On the other hand, biochemical pretreatment is highly selective in biomass deconstruction to their desired product formation. However, biochemical conversion first uses low-severity thermochemical pretreatment to partially break down the cell wall and expose the cellulose and hemicellulose fractions for improving enzyme accessibility. Elucidating the physicochemical effects of the possible pretreatments upon subsequent hydrolysis and fermentation of biomass has been a significant challenge.

Although several reviews have been present which describe the various categories of pretreatment processes individually, however, a comprehensive review covering different types of pretreatment processes along with their advantages and disadvantages was the need of the hour. Therefore, this review covers all the techniques that have been developed and used for pretreatment of lignocellulosic biomass, recent advancements in pretreatment technology, their mechanism of action, and effect on various lignocellulosic feedstocks.

## Methods of pretreatment

**Fig. 1:**
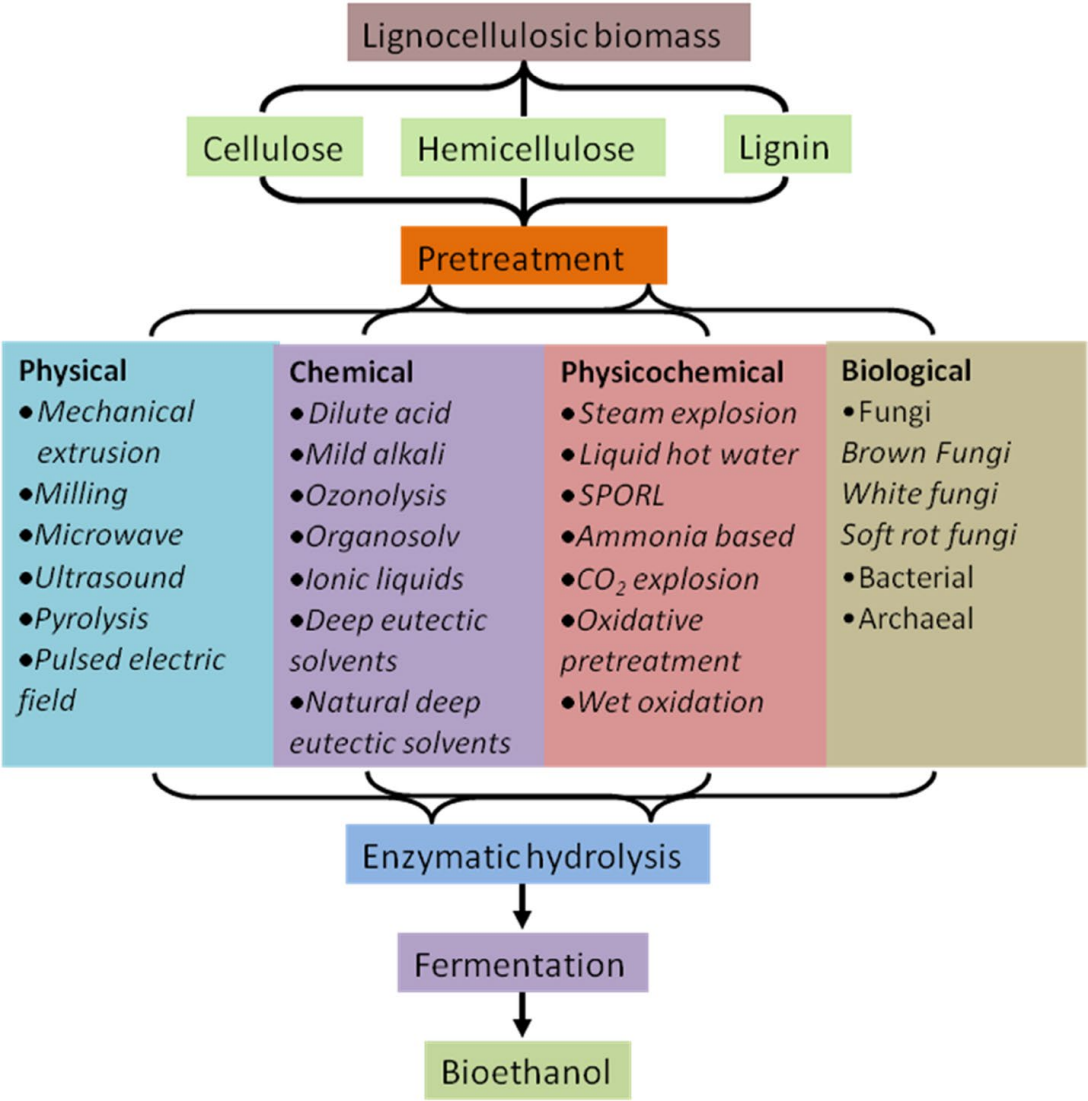
Overview of different pretreatment processes

The pretreatment of lignocellulosic feedstocks is an essential step and is required to alter the structure of biomass residues and expose the lignocellulosic fractions for easy access to enzymes during enzymatic hydrolysis and enhance the rate and yield of reducing sugars (Alvira et al. 2010). Basically, the pretreatment processes are classified into two major regimes viz. non-biological and biological. A list of promising and most commonly used pretreatment methods are listed in Fig. 1. Based on the type of the treatment process involved, lignocellulosic biomass pretreatment methods are broadly classified into two groups: Non-biological and biological. Non-biological pretreatment methods do not involve any microbial treatments and are roughly divided into different categories: physical, chemical, and physico-chemical methods. Here, we have reviewed the advances in few selective treatment methods that are most commonly employed in pretreatment process of a broad range of lignocellulosic feedstocks.

### Physical pretreatment

#### Mechanical extrusion

It is the most conventional method of biomass pretreatment where the feedstock materials are subjected to heating process (>300 °C) under shear mixing. This pretreatment process results mainly in production of gaseous products and char from the pretreated lignocellulosic biomass residues (Shafizadeh and Bradbury 1979). Due to the combined effects of high temperatures that are maintained in the barrel and the shearing force generated by the rotating screw blades, the amorphous and crystalline cellulose matrix in the biomass residues is disrupted. However, this method requires significant amount of high energy making it a cost intensive method and difficult to scale up for industrial purposes (Zhu and Pan 2010). Karunanithy et al. (2008) studied on the defibrillation and shortening of the biomass fibers and concomitant increase in the overall content of the carbohydrates and its availability for enzymatic hydrolysis process.

Zheng and Rehmann (2014) studied different process parameters of mechanical extraction process and found that the type of the screw design, compression ratio, screw speed, and barrel temperature affected the biomass pretreatment. Similarly, Karunanithy and Muthukumarappan (2010) also studied the effect of temperature and screw speed on pretreatment of corn cobs with different cellulose degrading enzymes and their ratios. When pretreatment was carried out at different temperatures (25, 50, 75, 100, and 125 °C) and different screw speeds (25, 50, 75, 100, and 125 rpm), maximum concentrations of glucose (75%), xylose (49%), and combined sugars (61%) were obtained at 75 rpm and 125 °C using cellulase and β-glucosidase in the ratio of 1:4, which were nearly 2.0, 1.7, and 2.0 times higher than the controls. These clearly indicated that optimization of the pretreatment process conditions and enzyme concentrations had a synergetic effect on the overall yields of reducing sugars.

Moreover, in another study, Karunanithy et al. (2013) selected different varieties of warm season grasses viz. switch grass, big bluestem, and prairie cord grass and studied the effect of different screw speeds (100, 150, and 200 rpm), barrel temperatures (50, 75, 100, 150, and 200 °C) and different concentrations of cellulase with β-glucosidase (1:1 to 1:4). In all the experiments, maximum reducing sugars were obtained when the ratio of cellulase and β-glucosidase was maintained at 1:4. The reducing sugar yields from the switchgrass pretreated at screw speed of 200 rpm and barrel temperature of 75 °C produced 28.2%, while big bluestem pretreated at screw speed of 200 rpm and 150 °C barrel temperature produced 66.2% and with prairie cord grass pretreated at 150 rpm and 100 °C produced 49.2%. Although the sugar yields are high, mechanical extrusion cannot alone suffice pretreatment of a range of lignocellulosic feedstocks with varied cellulose, hemicellulose, and lignin contents. Thus, it needs better pretreatment methods for higher sugar yields. Besides, sugar recovery is also significantly influenced by the properties of the biomass.

Karunanithy and Muthukumarappan (2010) studied the effect of varying moisture contents (15, 25, 35, and 45% wb) on the sugar recovery from switch grass and prairie cord grass at compression ratio (2:1 and 3:1), screw speed (50, 100 and 150 rpm), and barrel temperature (50, 100, and 150 °C). After enzymatic hydrolysis of the pretreated biomass, maximum 45.2% sugar was recovered from switch grass with 15% moisture content at screw speed of 50 rpm and barrel temperature of 150 °C, whereas a maximum of 65.8% sugar was recovered from prairie cord grass with 25% moisture content at screw speed of 50 rpm and barrel temperature of 50 °C. Alongside, low concentrations of glycerol and acetic acid in the range of 0.02–0.18 g/L were also produced. It is well known that glycerol and acetic acid are the byproducts that are formed during the pretreatment of lignocellulosic feedstocks. However, in this report, unlike hot compressed hot water and acid hydrolysis, the byproduct formation was significantly lower because in mechanical extraction only physical interactions were observed between the feedstock and the barrel blades. Similarly, Lamsal et al. (2010) also compared effects of grinding with extrusion on wheat bran and soybean hull. Better sugar yield was obtained in wheat bran through extrusion but not in soybean hulls. The most plausible reason could be due to the difference in the lignin contents between these biomass residues. Soybean hulls contain nearly twofold higher lignin content than the wheat bran. The residual high-lignin bound to the pretreated biomass could have shown a direct impact on the enzymatic hydrolysis. It is well known that the cellulose degrading enzymes avidly and irreversibly bind to lignin and thus not readily available for effective cellulose disruption. The combination of screw speed and barrel temperature maintained was 7 Hz/150 °C and 3.7 Hz/110 °C where highest sugar yield was produced.

Moreover, particle size of biomass plays an important role on the overall sugar recovery. Studies performed by Karunanithy and Muthukumarappan (2011) showed maximum sugar recovery from big blue stem obtained with 8-mm particle size, 20% wb moisture content at a barrel temperature of 180 °C with screw speed of 150 rpm, where 71.3% glucose, 78.5% xylose, and 56.9% combined sugars were obtained. While with switch grass, at similar particle size and moisture contents, but at a barrel temperature of 176 °C and screw speed of 155 rpm, maximum sugars of 41.4% glucose, 62.2% xylose, and 47.4% combined sugars were obtained. In another study, Zhang et al. (2012a, b) used a twin screw extruder for sugar recovery from corn stover. At 27.5% moisture content with a screw speed of 80 rpm and enzyme dose of 0.028 g enzyme/g dry biomass, glucose, xylose, and combined sugar recovery were 48.79, 24.98, and 40.07%, respectively. These were 2.2, 6.6, and 2.6 times more than that of untreated corn stover. Yoo (2011) compared a thermo-mechanical pretreatment process on soybean hulls. Under optimum processing conditions at screw speed of 350 rpm, barrel temperature of 80 °C and 40% moisture content, 95% cellulose was converted glucose. These above studies clearly demonstrate that mechanical extrusion treatment had a significant effect on breakdown of cellulose and hemicelluloses fractions from a wide variety of lignocellulosic feedstocks; however, when combined with other pretreatment methods, mechanical extrusion performs better and might enhance the overall yields of the reducing sugars.

#### Milling

Mechanical grinding (milling) is used for reducing the crystallinity of cellulose. It mostly includes chipping, grinding, and/or milling techniques. Chipping can reduce the biomass size to 10–30 mm only while grinding and milling can reduce the particle size up to 0.2 mm. However, studies found that further reduction of biomass particle below 0.4 mm has no significant effect on rate and yield of hydrolysis (Chang et al. 1997). Chipping reduces the heat and mass transfer limitations while grinding and milling effectively reduce the particle size and cellulose crystallinity due to the shear forces generated during milling. The type and duration of milling and also the kind of biomass determine the increase in specific surface area, final degree of polymerization, and the net reduction in cellulose crystallinity. Different milling methods viz. two-roll milling, hammer milling, colloid milling, and vibratory milling are used to improve the digestibility of the lignocellulosic materials (Taherzadeh and Karimi 2008). Compared to ordinary milling process, vibratory ball milling is found to be more effective in reducing cellulose crystallinity and improving the digestibility of spruce and aspen chips. Also, wet disk milling has been a popular mechanical pretreatment because of its low energy consumption. Disk milling enhances cellulose hydrolysis by producing fibers and is more effective as compared to hammer milling which produces finer bundles (Zhua et al. 2009). Hideno et al. (2009) compared the effect of wet disk milling and conventional ball milling pretreatment method over rice straw. The optimal conditions obtained were 60 min of milling in case of dry ball milling while 10 repeated milling operations were required in case of wet disk milling. Maximum glucose (89.4%) and xylose (54.3%) were obtained with conventional ball milling method as compared to 78.5% glucose and 41.5% xylose with wet disk milling method. However, wet disk milling had lower energy requirement, high effectiveness for enzymatic hydrolysis, and did not produce inhibitors. Lin et al. (2010) found wet milling better than dry milling for the pretreatment of corn stover. The optimum parameters for milling were particle size 0.5 mm, solid/liquid ratio of 1:10, 20 number of steel balls of 10 mm dia each, ball speed of 350 rpm/min grounded for 30 min. Better results were obtained when milling was combined with alkaline pretreatment method. As compared to wet milling process, alkaline milling treatment increased the enzymatic hydrolysis efficiency of corn stover by 110%. Sant Ana da Silva et al. (2010) performed a comparative analysis on effects of ball milling and wet disk milling on treating sugarcane bagasse and straw and found ball milling better pretreatment method than wet disk milling in terms of glucose and xylose hydrolysis yields. Ball milling-treated bagasse and straw produced 78.7 and 72.1 and 77.6 and 56.8%, glucose and xylose, respectively. Kim et al. (2013) compared three different milling methods i.e., ball, attrition, and planetary milling. Attrition and planetary mills were found more effective in reducing the size of biomass as compared to ball milling. Planetary mill produced highest amount of glucose and galactose than other milling methods tested. It is to be noted that all the mill pretreatment methods do not produce any toxic compounds like hydroxymethylfurfuraldehyde (HMF) and levulinic acid. This makes milling pretreatment a good choice of preliminary pretreatment method for a wide variety of lignocellulosic feed stocks. In another study, oil palm frond fiber when pretreated through ball mill produced glucose and xylose yields of 87 and 81.6%, respectively, while empty fruit bunch produced glucose and xylose yields of 70 and 82.3%, respectively (Zakaria et al. 2014).

#### Microwave

Microwave irradiation is a widely used method for lignocellulosic feedstock pretreatment because of various reasons such as (1) easy operation, (2) low energy requirement, (3) high heating capacity in short duration of time, (4) minimum generation of inhibitors, and (5) degrades structural organization of cellulose fraction. Moreover, addition of mild-alkali reagents is preferred for more effective breakdown. A study on microwave-based alkali pretreatment of switch grass yielded nearly 70–90% sugars (Hu and Wen 2008). Microwave-based alkali treatment of switchgrass and coastal bermudagrass using different alkalis found sodium hydroxide as the most suitable alkali. Under optimum conditions, switchgrass produced 82% glucose and 63% xylose while coastal bermudagrass produced 87% glucose and 59% xylose (Keshwani and Cheng 2010). Although not significant, the authors have correlated the differences in reducing sugars with the difference in the lignin content (19% in bermudagrass vs 22% in switchgrass) in these lignocellulosic feedstocks. Lu et al. (2011) studied microwave pretreatment of rape straw at different powers for different time durations. The higher power of microwave resulted in higher glucose production but treatment time did not have a significant effect at a specific power setting. Chen et al. (2011a, b) optimized the microwave heating at 190 °C for 5 min for bagasse pretreatment in terms of lignocellulosic structural disruption. In another investigation, Zhu et al. (2015a, b, 2016) have extensively studied the effects of microwave on chemically pretreated *Miscanthus*. Where, microwave treatment was applied to NaOH- and H_2_SO_4_-pretreated *Miscanthus* and found 12-times high sugar yield in half the time as compared to conventional heating NaOH and H_2_SO_4_ pretreatment. This was mainly due to the pre-disruption of crystalline cellulose and lignin solubilization with the chemical pretreatment. The maximum sugar yield obtained was 75.3% and glucose yield was 46.7% when pretreated with 0.2 M H_2_SO_4_ for 20 min at 180 °C. Similarly, Xu et al. (2011) developed an orthogonal design to optimize the microwave pretreatment of wheat straw and increased the ethanol yield from 2.678 to 14.8%. Bonmanumsin et al. (2012) reported substantial increase in yield of monomeric sugars from *Miscanthus sinensis* with microwave-assisted ammonium hydroxide treatment. Microwave pretreatment of oil palm empty fruit bunch fiber in the presence of alkaline conditions showed 74% reduction in lignin (Nomanbhay et al. 2013).

#### Ultrasound

Sonication is relatively a new technique used for the pretreatment of lignocellulosic biomass. However, studies in the laboratory have found sonication a feasible pretreatment option. Ultrasound waves produce both physical and chemical effects which alter the morphology of lignocellulosic biomass. Ultrasound treatment leads to formation of small cavitation bubbles which rupture the cellulose and hemicellulose fractions thereby increasing the accessibility to cellulose degrading enzymes for effective breakdown into simpler reducing sugars. Yachmenev et al. (2009) reported that the maximum cavitation was formed at 50 °C which is also the optimum temperature for many cellulose degrading enzymes. The ultrasonic field is primarily influenced by ultrasonic frequency and duration, reactor geometry and its type and solvent used. Furthermore, biomass characteristics, reactor configuration, and kinetics also influence the pretreatment through sonication (Bussemaker and Zhang 2013). Duration of sonication has maximum effect on pretreatment of biomass. However, prolonging sonication beyond a certain limit has no additional effect in terms of delignification and sugar release (Rehman et al. 2013). Sonication of corn starch slurry for 40 s increased the sugar yield by 5–6 times as compared to control (Montalbo et al. 2010). Sonication of alkaline pretreated wheat straw for 15–35 min increased delignification by 7.6–8.4% as compared to control (Sun and Tomkinson 2002). Besides duration, the frequency of sonication directly determines the power of sonication, which is also an important factor affecting the lignocellulosic feedstock pretreatment. Most of the researchers have used ultrasound frequency of 10–100 kHz for the pretreatment process which has been enough for cell breakage and polymer degradation (Gogate et al. 2011). However, higher sonication power level is reported to adversely affect the pretreatment process. High power leads to formation of bubbles near tip of ultrasound transducer which hinders the transfer of energy to the liquid medium (Gogate et al. 2011). Increased oxidation of cellulose has been observed in when the sonication power was increased to 400 W in 200 mL of slurry (Aimin et al. 2005). Similarly, poplar wood cellulose powder suspension turned viscous when treated with high power of 1200 W sonication (Chen et al. 2011a, b). Therefore, power and duration of sonication should be optimized based on the biomass and slurry characteristics to meet the desired pretreatment objectives.

#### Pyrolysis

Pyrolysis has also been employed for the pretreatment of lignocellulosic biomass, however, in biorefinery processes. Unlike bioethanol applications, pyrolysis treatment is used for production of bio-oil from lignocellulosic feedstocks. Although limited studies have been reported on use of pyrolysis for reducing sugars production, there are few reports on use of pyrolysis in pretreatment of chemically pretreated biomass. Hence, we have included a brief section on pyrolysis pretreatment in this review. Fan et al. (1987) applied mild acid hydrolysis (1 N sulfuric acid, at 97 °C for 2.5 h) on the pyrolysis-pretreated biomass and found ~85% conversion of cellulose to reducing sugars and >5% glucose. In brief, pyrolysis is a thermal degradation process where biomass was subjected to high-temperature treatment, generally operated at 500–800 °C in the absence of oxidizing agent. At this temperature, cellulose rapidly decomposes leading to formation of end products such as gaseous substances, pyrolysis oil, and charcoal (Kilzer and Broido 1965). Pyrolysis is divided into slow and fast pyrolysis based on the heating rate. The amount of each end product varies depending on the type of pyrolysis, biomass characteristics, and reaction parameters. Besides production of high value energy-rich products, pyrolysis is adapted by thermal industries due to easy transport management, storage, combustion, and retrofitting and is flexible in production and marketing. Pyrolysis is found to be more efficient when carried out in the presence of oxygen at lower temperatures (Shafizadeh and Bradbury 1979; Kumar et al. 2009). Shafizadeh and Bradbury carried out the pyrolysis in the presence of oxygen as well as nitrogen and found that a large number of bonds were broken in the presence of oxygen as compared to nitrogen. It was estimated that at 25 °C, 7.8 × 10^9^ bonds/min/g cellulose is cleaved in the presence of oxygen as compared to 1.7 × 10^8^ bonds with nitrogen under similar conditions.

Biomass to liquid (BtL) route is used for the production of transportation of fuels from biomass which includes conversion of biomass to syngas to high-quality Fischer–Tropsch (FT) fuels. Zwart et al. (2006) compared alternative BtL routes comprising chipping, torrefaction, pelletization, and pyrolysis. The most efficient and commercially feasible route was found to be based on torrefaction followed by pyrolysis and pelletization. The study also clearly demonstrated the advantage of pretreatment at the front end of BtL production route by decreasing the cost of FT product by ~3 Euro/GJ.

#### Pulsed-electric field

Pulsed-electric field (PEF) pretreatment exposes the cellulose present in the biomass by creating the pores in the cell membrane thereby allowing the entry of agents that will break the cellulose into constituent sugars. In PEF pretreatment, the biomass is subjected to a sudden burst of high voltage between 5.0–20.0 kV/cm for short durations (nano to milliseconds). The advantages of PEF are low energy requirement due to very short duration (100 μs) of pulse time and the treatment can be carried out at ambient conditions. Also, the PEF instrument is simple in design due to lack of moving parts (Kumar et al. 2009). Salerno et al. (2009) applied PEF to waste activated sludge and pig manure for increasing the production of methane during anaerobic digestion. Methane production increased twofold from sludge and 80% from pig manure as compared to untreated sludge and manure. Kumar et al. (2011) designed and developed a PEF system for the pretreatment of wood chip and switchgrass. They studied the effect of PEF on untreated and treated samples through the uptake of neutral red dye. Both switch grass and woodchip were found resistant to structural change at low field strengths. Switchgrass showed higher neutral red uptake at field strength ≥8 kV/cm while woodchip showed similar results at 10 kV/cm. Electric field strength and pulse duration are the two interdependent processing parameters affecting electroporation through PEF. Two different durations in the range of milliseconds and microseconds were applied to *Chlorella vulgaris* and found irreversible electroporation at >4 kV/cm in the millisecond range and at ≥10 kV/cm in the microseconds range (Luengo et al. 2015). Yu et al. (2016) optimized pressure, electric field strength, and pulse number on the juice expression yield, total polyphenols, and total proteins content in the expressed juices of rapeseed stem biomass. The optimum conditions of electric field strength *E* = 8 kV/cm, pressure *P* = 10 bar and pulse number tPEF = 2 ms increased juice yield from 34 to 81%. Total polyphenols and total proteins content increased significantly after PEF pretreatment.

### Chemical pretreatment

#### Dilute acid

Although acid treatment is the most commonly used conventional pretreatment method of lignocellulosic feedstocks, it is less attractive due to the generation of high amount of inhibitory products such as furfurals, 5-hydroxymethylfurfural, phenolic acids, and aldehydes. The corrosive and toxic nature of most acids requires a suitable material for building the reactor which can sustain the required experimental conditions and corrosive nature of acids (Saha et al. 2005). Still it is the most widely employed pretreatment method on industrial scale. Based on the type of end application, two types of acid pretreatments are developed; high temperature (above 180 °C) for short duration (1–5 min) and low temperature (<120 °C) for long duration (30–90 min), respectively. In some cases, enzymatic hydrolysis step could easily be avoided as acid itself hydrolyses the biomass into fermentable sugars. However, extensive washing is necessary to remove acid before fermentation of sugars (Sassner et al. 2008). Different types of reactors such as percolation, plug flow, shrinking-bed, batch, flow-through, and counter current reactors have been developed. However acid treatment generates inhibitors which need to be removed before further processing. Also, the concentrated acid must be recovered after hydrolysis in order to make the process economically feasible. Different acids have been used for the pretreatment of a variety of biomass. Some of the commonly used acids are discussed here:

##### Sulfuric acid

The most common commercially used acid is dilute sulphuric acid (H_2_SO_4_). It has been widely used to pretreat switchgrass (Digman et al. 2010), corn stover (Xu et al. 2009), spruce (Shuai et al. 2010), and poplar (Kumar and Wyman 2009). Pretreatment of bermuda grass and rye straw with 1.5% sulphuric acid followed by enzymatic hydrolysis yielded 19.71 and 22.93% reducing sugars from bermuda grass and rye straw, respectively (Sun and Cheng 2005). Kim et al. (2011a, b) carried pretreatment of rice straw in two-stage process using aqueous ammonia and dilute H_2_SO_4_ in percolation mode. The yield of reducing sugars was observed to be 96.9 and 90.8%, respectively, indicating that combination of these two processes resulted in better removal of lignin and hemicelluloses. Pretreatment liquor of *Eulaliopsis binata* (a perennial grass commonly found in India and China) with diluted H_2_SO_4_ at optimum conditions resulted in 21.02% total sugars, 3.22% lignin, and 3.34% acetic acid with the generation of low levels of inhibitors (Tang et al. 2013). Acid pretreatment of wheat and rice straw gave maximum sugar yield of 565 and 287 mg/g, respectively, with no furfural and hydroxymethyl furfural formation (Saha et al. 2005).

Due to its low cost, pretreatment of lignocellulosic biomass through sulfuric acid is a conventional method. However, it has certain disadvantages such as production of inhibitory compounds and corrosion of reaction vessel (Lee and Jeffries 2011). Therefore researchers have carried out the pretreatment of lignocellulosic biomass through various other acids such as oxalic acid and maleic acid which are discussed later in the review (Kootstra et al. 2009; Lu and Mosier 2007; Lee et al. 2009).

##### Dicarboxylic acids: oxalic and maleic acid

As described earlier, other class of acids called as dicarboxylic acids are being tested by researchers in order to overcome the drawbacks associated with sulfuric acid. Such acids have higher pKa values than sulfuric acid and therefore have a higher solution pH as compared to sulfuric acid which is a type of mineral acid. Dicarboxylic organic acids exhibit two pKa values which make them more efficient for carrying out the hydrolysis of the substrate over a range of temperature and pH values (Lee and Jeffries 2011).

Apart from above mentioned advantages, oxalic acid is less toxic to yeasts and other microorganisms than sulfuric and acetic acids, does not hamper glycolysis and does not produce odor. Lee and coworkers (2011) used oxalic acid for the pretreatment of corn cobs. Corn cob was heated to 168 °C and kept for 26 min. A total sugar yield of 13% was obtained through oxalic acid pretreatment. Also, it produced very less amount of inhibitors.

Maleic acid is another common dicarboxylic acid used for the pretreatment purpose. Along with the advantages mentioned above, maleic acid in particular has k_hyd/_k_deg_ which favors cellulose hydrolysis to glucose over glucose degradation (Mosier et al. 2002). Lee and Jeffries (2011) investigated the effects of oxalic, maleic, and sulfuric acid on hydrolysis and degradation of lignocellulosic biomass at same combined severity factor (CSF) during hydrolysis. At low CSF values, xylose and glucose concentrations were found to be highest in maleic acid followed by oxalic acid and sulfuric acid. The subsequent fermentation with pretreated biomass yielded maximum ethanol (19.2 g/L) at CSF 1.9 when maleic acid was used for pretreatment of biomass.

Marzialetti et al. (2008) studied the effect of different acids viz. TFA, HCl, H_2_SO_4_, HNO_3_, and H_3_PO_4_ on loblolly pine in a batch reactor. TFA yielded highest amount of soluble monosaccharides at 150 °C and pH 1.65.

#### Mild-alkali

In contrary to acid treatment, alkali pretreatment methods are in general performed at ambient temperature and pressure. The most commonly used alkali reagents are the hydroxyl derivatives of sodium, potassium, calcium, and ammonium salts. Among these hydroxyl derivatives, sodium hydroxide was found to be most effective (Kumar and Wyman 2009). Alkali reagents degrade the side chains of esters and glycosides leading to structural modification of lignin, cellulose swelling, cellulose decrystallization, and hemicellulose solvation (Cheng et al. 2010; Ibrahim et al. 2011; McIntosh and Vancov 2010; Sills and Gossett 2011). Sun et al. (1995) optimized the concentration, temperature, and duration of pretreatment using sodium hydroxide. The optimized condition was 1.5% sodium hydroxide at 20 °C for 144 h released 60% lignin and 80% hemicellulose. Zhao et al. (2008) showed the effect of sodium hydroxide on different biomass viz. wheat straw, hardwoods, switchgrass, and softwoods containing less than 26% lignin. However, no effect of dilute NaOH was observed on softwoods with lignin content greater than 26% (Kumar and Wyman 2009). As compared to untreated cellulose, the sodium hydroxide treated corn stover showed increase in biogas production by 37% (Zhu et al. 2010). As compared to acid pretreatment, the solubility of cellulose and hemicellulose is very low with the alkali pretreatment. The solubility improves on increasing the internal surface area of cellulose, decreasing the degree of polymerization and crystallinity, and disrupting the lignin structure (Taherzadeh and Karimi 2008). The conditions for mild alkali pretreatment are less harsh as compared to other pretreatment methods especially acid pretreatment method. Mild alkali pretreatment can be successfully carried out at ambient conditions, however, higher temperature are required if the pretreatment is needed to be carried out for longer duration. Further, a neutralizing step is required to remove the inhibitors as well as lignin (Brodeur et al. 2011). The benefit of lime pretreatment is the low cost of lime as compared to other alkaline agents. For example, in 2005, cost of hydrated lime was $70/ton as compared to $270/ton ammonia and $320/ton for 50 wt% NaOH and 45 wt% KOH (Brodeur et al. 2011). Also, it can be easily recovered from hydrolysate by reaction with CO_2_. Park et al. (2010a, b) modified the lime pretreatment method by neutralizing the lime with carbon dioxide before hydrolysis. This eliminated the solid–liquid separation step resulting in 89% glucose recovery from leafstar rice straw. They also applied this modification to examine simultaneous saccharification and fermentation (SSF) by using *Saccharomyces cerevisae* and *Pichia stipitis* which found 74% increase in ethanol yield after 79 h of fermentation at 30 °C. Being an inexpensive pretreatment method, the only drawback of alkali treatment is its high downstream processing cost because the process utilizes a large quantity of water for removing the salts from the biomass and is a cumbersome process to remove them.

#### Ozonolysis

Ozone treatment is mainly used for reducing the lignin content of lignocellulosic biomass as it mainly degrades lignin but negligibly affects hemicellulose and cellulose (Kumar et al. 2009). It has been used for removal of lignin in various biomass such as wheat straw (Ben and Miron 1981), bagasse, green hay, peanut, pine (Neely 1984) and poplar sawdust (Vidal and Molinier 1988). A laboratory scale ozonolysis setup was designed and developed by Vidal and Molinier for the pretreatment of different biomass. The pretreatment of wheat straw in the mentioned reactor resulted in 60% removal of lignin followed by fivefold increase in enzymatic hydrolysis. In case of poplar sawdust, lignin percentage was reduced to 8% and sugar yield increased to 57% (Vidal and Molinier 1988). Unlike other chemical pretreatment methods, ozonolysis is performed at ambient temperature and pressure. Also, it does not produce any toxic inhibitors therefore is environment friendly and does not affect the post-pretreatment processes like enzymatic hydrolysis and yeast fermentations (Quesada et al. 1999). The important factor which affects the ozone pretreatment is the moisture content of the biomass, higher the moisture content, lower the lignin oxidization. Although ozonolysis is an effective pretreatment method, the high amount of ozone required makes it an expensive pretreatment method, making it a less suitable option for pretreatment at industrial scale. In order to make an economically viable pretreatment method, research is in progress in different areas such as generation of industrially feasible ozone concentrations, development of reactors such as packed bed, fixed-bed, and stirred tank semi-batch reactors that are capable of accommodating large quantities of low-moisture (<30%) biomass residues having particle size between 1 and 200 mm.

#### Organosolv

This process involves addition of aqueous organic solvents such as ethanol, methanol, ethylene glycol, acetone etc. to the biomass under specific condition of temperature and pressure (Alriols et al. 2009; Ichwan and Son 2011). Commonly, this process takes place in the presence of an acid, base or salt catalyst (Bajpai 2016). Temperature in organosolv pretreatment depends on the type of biomass and catalyst involved and may reach up to 200 °C. This process is mainly used for the extraction of lignin which is a value-added product. Apart from lignin, cellulose fraction and hemicellulose syrup of C5 and C6 sugars are also produced during the course of organosolv pretreatment. Removal of lignin from the biomass exposes the cellulose fibers for enzymatic hydrolysis leading to higher conversion of biomass (Agbor et al. 2011). The physical characteristics of pretreated biomass such as fiber length, degree of cellulose polymerization, crystallinity etc. depends upon variable factors such as temperature, reaction time, solvent concentration and catalyst used. High temperatures, high acid concentrations, and long reaction time have led to the formation of inhibitors of fermentation. Park et al. (2010a, b) studied the effect of different catalysts (H_2_SO_4_, NaOH, and MgSO_4_) on pine and found H_2_SO_4_ as the most effective catalyst in terms of ethanol yield. However, in terms of digestibility, NaOH was found to be effective when its concentration was increased by 2%. H_2_SO_4_ has high reactivity therefore has proven to be a very strong catalyst but at the same time it is toxic, corrosive and is inhibitory in nature. The main drawback of this process is the high cost of the solvents, though this drawback can be minimized by recovering and recycling solvents through evaporation and condensation. Removal of solvents is very important because the solvent may cause negative effect on growth of microorganisms, enzymatic hydrolysis, and fermentation (Agbor et al. 2011). Also, organosolv is less preferred due to high risk involved in handling harsh organic solvents that are highly flammable. In absence of proper safety measures, it can cause severe damage leading to large fire explosions. The Battelle is a type of organosolv method that treats the biomass with mixture of phenol, HCl, and water at temperature 100 °C and pressure 1 atm (Villaverde et al. 2010). Acid is responsible for the depolymerization of lignin as well as it hydrolyses the hemicellulose portion of the biomass. Lignin is dissolved in the phenol while the sugars (monosaccharides) are found in the aqueous phase upon cooling. Likewise formasolv is a type of organosolv involving formic acid, water, and HCl. Lignin is soluble in formic acid and the pretreatment process can be carried out at low temperature and pressure (Zhao and Liu 2012). Unlike formasolv, ethanosolv (involving ethanol) is operated at high temperature and pressure and recovers value-added products namely cellulose, hemicellulose, and pure lignin. Further purification may be carried out through ionic liquids which are discussed later in this review (Prado et al. 2012). The less toxic nature of ethanol as compared to methanol and it being the final product makes it more popular as compared to other solvents (Kim et al. 2011a, b). However, presence of ethanol inhibits the performance of hydrolytic enzymes, therefore lower ethanol: water is used for hydrolysis of hemicellulose and enzymatic degradation of pretreated biomass (Huijgen et al. 2008). Also, nearly complete recovery of ethanol and water is a major advantage which reduces the operation cost (Koo et al. 2012; Alriols et al. 2010). Mesa et al. (2011) applied ethanosolv on sugarcane bagasse for production of reducing sugars. 29.1% reducing sugars were produced by 30% ethanol for 60 min at 195 °C. Similarly, horticultural waste was pretreated by a modified method using ethanol under mild conditions for bioethanol production. Pretreatment resulted in hydrolysate containing 15.4% reducing sugar after 72 h, which after fermentation produced 1.169% ethanol in 8 h using *Saccharomyces cerevicae* (Geng et al. 2012). Hideno et al. (2013) reported utilization of alcohol-based organosolv treatment in combination with ball milling for pretreatment of Japanese cypress (*Chamaecyparis obtusa*). They observed that combination of alcohol-based organosolv treatment in mild conditions and short time ball milling had a synergistic effect on the enzymatic digestibility. Ichwan and Son (2011) studied the effect of various solvents such as ethanol–water, ethylene glycol–water, and acetic acid–water mixture to extract cellulose from oil palm pulp. The yield of organosolv pulping with ethylene glycol–water, ethanol–water, and acetic acid–water mixture was 50.1, 48.1, and 41.7%, respectively. Panagiotopoulos et al. (2012) treated poplar wood chips with steam followed by organosolv treatment for separating hemicellulose, lignin, and cellulose components. Lignin extraction was found to increase to 66%, while 98% of the cellulose was recovered by two stage pretreatment process and 88% of cellulose was hydrolyzed to glucose after 72 h.

#### Ionic liquids

Ionic liquids have received great attention in last decade for the pretreatment of lignocellulosic biomass. Ionic liquids are comparatively a new class of solvents which are entirely made of ions (cations and anions), have low melting points (<100 °C), negligible vapor pressure, high thermal stabilities, and high polarities (Zavrel et al. 2009; Behera et al. 2014). Imidazolium salts are the most commonly used ILs. ILs are assumed to compete with lignocellulosic components for hydrogen bonding there by disrupting its network (Moultrop et al. 2005). Table 2 lists various ionic liquids used for the treatment of a variety of biomasses.

**Table 2:** Different types of ionic liquids applied for the pretreatment of different biomass (Bajpai 2016)

Biomass	Ionic liquid	Abbreviated symbol
Poplar wood	1-Ethyl-3-methylimidazolium diethyl phosphate-acetate	Emim-Ac
Pine	1-Allyl-3-methylimidazolium chloride	Amim-Cl
Eucalyptus	Ethyl-3-methylimidazolium diethyl phosphate-acetate	Emim-Ac
Spruce	1-Allyl-3-methylimidazolium chloride	Amim-Cl
Bagasse	Ethyl-3-methylimidazolium diethyl phosphate-acetate	Emim-Ac
Switch grass	Ethyl-3-methylimidazolium diethyl phosphate-acetate	Emim-Ac
Bamboo	1-Ethyl-3-methylimidazolium diethyl phosphate-glycine	Emim-Gly
Wheat straw	1-Allyl-3-methylimidazolium chloride and chloride	Amim-Ac
	1-Butyl-3-methylimidazolium-acetate	Bmim-Ac
Water hyacinth	1-Butyl-3-methylimidazolium-acetate	Bmim-Ac
Rice husk	1-Butyl-3-methylimidazolium-chloride	Bmim-Cl
	1-Ethyl-3-methylimidazolium diethyl phosphate-acetate	Emim-Ac
Rice straw	Cholinium amino acids	Ch-Aa
Kenaf powder	Cholinium acetate	Ch-Ac

According to Li et al. (2011), with suitable selection of anti-solvents up to 80% lignin and hemicellulose can be fractionated. Dadi et al. (2006) used 1-butyl-3-methylimidazolium chloride (Bmim-Cl) for pretreatment of Avicel—PH-101 reported 50- and 2-fold increase in enzymatic hydrolysis rate and yield, respectively, as compared to untreated Avicel. Liu and Chen (2006) used Bmim-Cl for pretreating wheat straw and found significant improvement in enzymatic hydrolysis yield. They found that Bmim-Cl modified structure of wheat straw by reducing the polymerization and crystallinity and solubilizing cellulose and hemicellulose. Sugarcane bagasse pretreated with 3-N-methylmorpholine-N-oxide (NMMO) showed twofold increase in enzymatic hydrolysis yield as compared to untreated bagasse (Kuo and Lee 2009). Ionic liquids has been able to effectively pretreat lignocellulosic biomass, however, there are certain challenges that need to be addressed such as high cost of ILs, difficulty in recycling and reuse, inhibitor generation etc.

#### Deep eutectic solvents

These are relatively a new class of solvents having many characteristics similar to ionic liquids. A deep eutectic solvent (DES) is a fluid generally composed of two or three cheap and safe components that are capable of self-association, often through hydrogen bond interactions, to form a eutectic mixture with a melting point lower than that of each individual component (Zhang et al. 2012a, b). These DES were able to solve some of the key concerns associated with ILs. Deep eutectic solvents can be described by the general formula

$${\text{Cat}}^{ + } {\text{X}}^{ - } z{\text{Y}}$$

where Cat^+^ is in principle any ammonium, phosphonium, or sulfonium cation, and X is a Lewis base, generally a halide anion. The complex anionic species are formed between X^−^ and either a Lewis or Brønsted acid Y (*z* refers to the number of Y molecules that interact with the anion) (Smith et al. 2014). Most of the DESs have used choline chloride (ChCl) as hydrogen bond acceptor. ChCl is low-cost, biodegradable, and non-toxic ammonium salt which can be extracted from biomass. ChCl is able to synthesize DESs with hydrogen donors such as urea, carboxylic acids, and polyols. Although DESs are similar to ILs in terms of physical behavior and physical properties, DESs cannot be considered as ionic liquids due to the fact that DESs are not entirely composed of ionic species and can be obtained from non-ionic species (Zhang et al. 2012a, b).

#### Natural deep eutectic solvents

In the recent past, a large number of natural products have been brought into the range of ILs and DES. These products include choline, urea, sugars, amino acids, and several other organic acids (Dai et al. 2013). Such solvents obtained from natural sources are termed as Natural Deep Eutectic Solvents (NADES). Unlike ILs, NADES are cost effective, easier to synthesize, non-toxic, biocompatible, and highly biodegradable. Moreover, many studies recovered and reused these novel solvents with high efficiency. NADES are prepared by the complex formation between a hydrogen acceptor and a hydrogen bond donor. The decrease in melting point of the prepared solvent mixtures is due to the charge delocalization of the raw individual components. Foreseeing the potentiality of NADES in diverse applications, these solvents are regarded as the solvents for the twenty first century (Paiva et al. 2014). Moreover, recent research on lignocellulosic feedstock pretreatment with NADES reagents showed high specificity towards lignin solubilisation and extraction of high purity lignin from agricultural residue such as rice straw (Kumar et al. 2016). Despite having a lot of potential for the extraction of natural products, the high viscosity of NADES is an obvious disadvantage. Dai et al. (2015) studied the dilution effect on the physicochemical properties of NADES. FT-IR and ^1^H NMR studies showed intense H-bonds between the two components of NADES system. However, the dilution with water weakened the interactions. At around 50% (v/v) dilution with water, the intense hydrogen interactions disappeared completely. The viscosity of NADES reduced to the order of water and conductivity increased up to 100 times for some NADES reagents. Along with pretreatment, NADES can prove to be a game changer concept in pharma, food processing, and enzyme industries.

### Physico-chemical pretreatment

#### Steam explosion

Steam pretreatment is one of the most commonly used physicochemical methods for pretreatment of lignocellulosic biomass. Earlier, this method was known as steam explosion because of the belief that an explosive action on the biomass was required to prepare them for hydrolysis (Agbor et al. 2011). Due to the changes that occur during this process, this method is also called ‘auto hydrolysis.’ Steam pretreatment is typically a combination of mechanical forces (pressure drop) and chemical effects (autohydrolysis of acetyl groups of hemicellulose). In this process, biomass is subjected to high pressure (0.7–4.8 MPa) saturated steam at elevated temperatures (between 160 and 260 °C) for few seconds to minutes which causes hydrolysis and release of hemicellulose. The steam enters the biomass expanding the walls of fibers leading to partial hydrolysis and increasing the accessibility of enzymes for cellulose. After this the pressure is reduced to atmospheric condition (Rabemanolontsoa and Saka 2016). During this pretreatment, the hydrolysis of hemicellulose into glucose and xylose monomers is carried out by the acetic acid produced from the acetyl groups of hemicellulose; hence this process is also termed as autohydrolysis (Mosier et al. 2005). The factors that affect steam pretreatment are temperature, residence time, biomass size, and moisture content (Rabemanolontsoa and Saka 2016). Wright (1988) found low temperature and longer residence time (190 °C for 10 min) better as compared to high temperature and lower residence time (270 °C for 1 min) due to less fermentation inhibitory product formation in the earlier process. Several biomasses have shown positive effects on pretreatment with steam such as poplar wood (*Populus tremuloides*) (Grous et al. 1986), pine chips, French maritime pine (*Pinus pinaster*), rice straw, bagasse, olive stones, giant miscanthus (*Miscanthus giganteus*), and spent Shiitake mushroom media (Jacquet et al. 2012). The efficiency of steam pretreatment can be effectively enhanced in the presence of catalysts such as H_2_SO_4_, CO_2_ or SO_2_. Out of these catalysts, acid catalyst has been found to most successful in terms of hemicellulose sugar recovery, decreased production of inhibitory compounds and improved enzymatic hydrolysis. Steam pretreatment is found to be effective for the pretreatment of hardwoods and agricultural residues, though acid catalyst is added in case of soft woods for effective pretreatment. Limited use of chemicals, low energy requirement, no recycling cost and environment friendly are some of the advantages of steam pretreatment method. On the other hand, the possibility of formation of fermentation inhibitors at high temperature, incomplete digestion of lignin-carbohydrate matrix and the need to wash the hydrolysate which decreases the sugar yield by 20% are few disadvantages associated with steam pretreatment (Agbor et al. 2011).

#### Liquid hot water

This method, also called as hot compressed water is similar to steam pretreatment method but as the name suggests, it uses water at high temperature (170–230 °C) and pressure (up to 5 MPa) instead of steam. This leads to hydrolysis of hemicellulose and removes lignin making cellulose more accessible. This also avoids the formation of fermentation inhibitors at high temperatures (Yang and Wyman 2004). Different researchers have described liquid hot water (LHW) with different terms such as solvolysis, hydrothermolysis, aqueous fractionation and aquasolv (Agbor et al. 2011). LHW can be performed in three different ways based on the direction of flow of water and biomass into reactor. (1) Co-current pretreatment, in which both the slurry of biomass and the water is heated to the required temperature and held at the pretreatment conditions for controlled residence time before being cooled. (2) Counter current pretreatment, in which the hot water is pumped against the biomass in controlled conditions. (3) Flow through pretreatment where the biomass acts like a stationary bed and hot water flows through the biomass and the hydrolyzed fractions are carried out of the reactor. Abdullah and coworkers (2014) conducted studies on LHW to investigate the hydrolysis performance. The optimization could not be carried out at same severity due to the difference in rate of hydrolysis of cellulose and hemicellulose. Therefore, a two-step hot compressed water treatment was proposed. First stage is carried out at low severity for hydrolyzing the hemicellulose while second stage is carried out at high severity for depolymerization of cellulose and increase sugar yield. Ogura et al. (2013) and Phaiboonsilpa (2010) applied two-step hydrolysis (I step: 230 °C-10 MPa-15 min; II step: 275 °C-10 MPa-15 min) to Japanese beech, Japanese cedar, Nipa frond and rice straw and found to solubilize 92.2, 82.3, 92.4, and 97.9% of the starting biomass, respectively. This has proved that LHW is capable of acting on a large variety of biomass including softwoods (Rabemanolontsoa and Saka 2016). Low-temperature requirement, minimum formation of inhibitory compounds and low cost of the solvent are some of the advantages associated with LHW. However, it requires large amount of energy in downstream processing due to large amount of water involved (Agbor et al. 2011).

#### Wet oxidation

Wet oxidation is one of the simple methods of lignocellulosic pretreatment where the air/oxygen along with water or hydrogen peroxide is treated with the biomass at high temperatures (above 120 °C for 30 min) (Varga et al. 2003). Earlier this method as also used for waste water treatment and soil remediation (Chaturvedi and Verma 2013). This method is most suitable for lignin enriched biomass residues. The efficiency of wet oxidation is dependent on three factors: oxygen pressure, temperature, and reaction time. In this process, when the temperature is raised above 170 °C, water behaves like an acid and catalyzes hydrolytic reactions. The hemicelluloses are broken down into smaller pentose monomers and the lignin undergoes oxidation, while the cellulose is least affected by wet oxidation pretreatment. Besides these, reports on addition of chemical agents like sodium carbonate and alkaline peroxide in wet oxidation reduced the reaction temperature, improved hemicellulose degradation and decreased the formation of inhibitory components such as furfurals and furfuraldehydes (Banerjee et al. 2011). This pretreatment method is unlikely to reach industrial scale of biomass pretreatment because of the high cost of the hydrogen peroxide and the combustible nature of the pure oxygen (Bajpai 2016). Szijártó et al. (2009) applied wet oxidation for the pretreatment of common reed (*Phragmites australis*). The treatment resulted in three fold increase in digestibility of reed cellulose by cellulase as compared to control. 51.7% of hemicellulose and 58.3% of lignin was solubilised and 82.4% of cellulose got converted into cellulose on enzymatic hydrolysis of the pretreated fibers. The pretreated fibers produced 0.87% ethanol when underwent simultaneous saccharification and fermentation. Banerjee et al. (2009) optimized the wet oxidation conditions for rice husk for the production of ethanol. The optimized conditions of 0.5 MPa pressure, 185 °C temperature for 15 min yielded 67% of cellulose, removed 89% lignin, and solubilised 70% hemicellulose. Reducing sugar yields up to 70% have been obtained by utilizing this pretreatment process. Alkaline Peroxide-Assisted Wet Air Oxidation (APAWAO) treatment on rice husk resulted in solubilization of 67 and 88 wt% of hemicellulose and lignin, respectively. The glucose amount increased 13-fold as compared from untreated rice husk (Banerjee et al. 2011). This pretreatment method is unlikely to reach industrial scale of biomass pretreatment because of the high cost of the hydrogen peroxide and the combustible nature of the pure oxygen (Bajpai 2016).

#### SPORL treatment

Sulfite pretreatment to overcome recalcitrance of lignocellulose (SPORL) is a popular and efficient pretreatment method for lignocellulosic biomass (Xu et al. 2016). It is carried out in a combination of two steps: First, the biomass is treated with calcium or magnesium sulfite to remove hemicellulose and lignin fractions. In the second step, the size of the pretreated biomass is reduced significantly using mechanical disk miller. Zhu et al. (2009) studied the effect of SPORL pretreatment on spruce chips using 8–10% bisulfite and 1.8–3.7% sulfuric acid at 180 °C for 30 min. After 48 h of hydrolysis with 14.6 FPU cellulase +22.5 CBU β-glucosidase per gram of substrate, more than 90% substrate was converted to cellulose. Also, only 0.5% hydroxymethyl furfural (HMF) and 0.1% furfural (fermentation inhibitors), respectively, were formed as compared to 5% HMF and 2.5% furfural formation during the acid catalyzed steam pretreatment of spruce. The amount of HMF and furfural was also reported to decrease with increasing bisulfite. The possible reason is that at same acid charge, higher amount of bisulfite leads to higher pH which reduces the decomposition of sugars to HMF and furfural.

SPORL pretreatment on switchgrass was carried out by Zhang et al. (2013) with temperature ranging between 163 and 197 °C for a period ranging from 3–37 min with sulfuric acid dosage (0.8–4.2%) and sodium sulfite dosage (0.6–7.4%). The results found improved digestibility of switchgrass by removing hemicellulose, dissolving lignin partially and decreasing hydrophobicity of lignin by sulfonation. SPORL pretreated switchgrass was hydrolysed by 83% in 48 h with 15 FPU cellulase and 30 CBU β-glucosidase/g cellulose. SPORL pretreatment method when compared with dilute acid and alkali pretreatments, was found to give the highest substrate yield of 77.2% as compared to 68.1 and 66.6% by dilute acid and alkali pretreatment, respectively. Sodium sulfide and sodium sulfite along with sodium hydroxide were applied for pretreatment of corncob, bagasse, water hyacinth and rice husk. Pretreatment under optimized conditions yielded 97% lignin and 93% hemicellulose from water hyacinth and rice husk, and 75% lignin and 90% hemicellulose were removed from bagasse and rice husk (Idrees et al. 2013).

SPORL pretreatment has been popular in the recent times because of its versatility, efficiency, and simplicity. It reduces the energy consumption to 1/10 required for the reduction of size of biomass. It has very high conversion rate of cellulose to glucose and maximizes hemicellulose and lignin removal and recovery. It has the capacity to process a variety of biomass and has excellent scalability for commercial production by retrofitting into existing mills for production of biofuels. However, certain issues such as sugar degradation, requirement of large volumes of water for post-pretreatment washing and high cost of recovering pretreatment chemicals need to be addressed for making SPORL a cost effective pretreatment technology (Bajpai 2016).

#### Ammonia-based pretreatment

Methods that use liquid ammonia for the pretreatment of lignocellulosic biomass are Ammonia fiber explosion (AFEX), Ammonia recycle percolation (ARP) and soaking aqueous ammonia (SAA). AFEX is conducted at ambient temperature while ARP is conducted at high temperatures (Agbor et al. 2011) SAA is a form of AFEX which treats biomass through aqueous ammonia in a batch reactor at 30–60 °C which decreases the liquid through-put during process of pretreatment (Kim and Lee 2005a). In AFEX, lignocellulosic biomass is heated with liquid ammonia (in 1:1 ratio) in a closed vessel at temperature 60–90 °C and pressure above 3 MPa for 30–60 min. After holding the desired temperature in vessel for 5 min, valve is opened which explosively releases the pressure leading to evaporation of ammonia and drop in temperature of the system (Alizadeh et al. 2005). It is similar to steam explosion but ammonia is used instead of water (Rabemanolontsoa and Saka 2016). Lignocellulosic biomass when treated with ammonia at high pressure and given temperature causes swelling and phase change in cellulose crystallinity of biomass leading to increase in the reactivity of leftover carbohydrates after pretreatment. The lignin structure gets modified which increases the water holding capacity and digestibility. Unlike other pretreatment methods, AFEX treatment does not produce inhibitors, which is highly desirable for downstream processing. Besides, the overall cost of the pretreatment process is significantly low due to the absence of additional steps like water washing, detoxification, recovery, and reuse of large quantities of water. More than 90% of celluloses and hemicelluloses could be converted to fermentable sugars if pretreated with AFEX under optimized conditions of ammonia loading, temperature, pressure, moisture content and pretreatment time (Uppugundla et al. 2014). Moreover, the ammonia could be recovered and recycled to decrease the overall cost of the pretreatment process.

Another process that utilizes ammonia is ammonia recycle percolation (ARP). In this process, aqueous ammonia (5–15 wt%) is passed through a reactor containing biomass. The temperature range is between 140 and 210 °C with a reaction time of 90 min and percolation rate is 5 mL/min after which the ammonia is recycled (Sun and Cheng 2002; Kim et al. 2008). ARP is capable of solubilizing hemicellulose but cellulose remains unaffected (Alvira et al. 2010). The disadvantage with ARP is high requirement of energy to maintain process temperature. Both AFEX and ARP have been found to effective for herbaceous plants, agricultural residues and MSW. ARP pretreatment is found effective for hardwoods also (Kim and Lee 2005b). Another technology soaking aqueous ammonia (SAA) requires less energy as it is performed at low temperature (30–75 °C).

#### CO_2_ explosion

This process carries out the pretreatment of biomass through supercritical CO_2_ which means the gas behaves like a solvent. The supercritical CO_2_ is passed through a high pressure vessel containing the biomass (Kim and Hong 2001). The vessel is heated to the required temperature and kept for several minutes at high temperatures (Hendricks and Zeeman 2009). CO_2_ enters the biomass at high pressure and forms carbonic acid which hydrolyses the hemicellulose. The pressurized gas when released disrupts the biomass structure which increases the accessible surface area (Zheng et al. 1995). This pretreatment method is not suitable for biomass having no moisture content. Higher the moisture content in the biomass, higher the hydrolytic yield (Kim and Hong 2001). Low cost of carbon dioxide, low temperature requirement, high solid capacity, and no toxin formation makes it an attractive process. However, high cost of reactor which can tolerate high pressure conditions is a big obstacle in its application on large scale (Agbor et al. 2011).

#### Oxidative pretreatment

It involves treatment of lignocellulosic biomass by oxidizing agents such as hydrogen peroxide, ozone, oxygen or air (Nakamura et al. 2004). A number of chemical reactions such as electrophilic substitution, side chain displacements, and oxidative cleavage of aromatic ring ether linkages may take place during oxidative pretreatment. This process causes delignification by converting lignin to acids, which may act as inhibitors. Therefore, these acids need to be removed (Alvira et al. 2010). A major drawback of oxidative pretreatment is that it damages a significant amount of hemicellulose making it unavailable for fermentation (Lucas et al. 2012). The most commonly employed oxidizing agent is hydrogen peroxide. It has been found that hydrolysis of hydrogen peroxide leads to formation of hydroxyl radicals which are responsible for degradation of lignin and production of low molecular weight products. Removal of lignin from lignocellulose exposes cellulose and hemicellulose leading to increased enzymatic hydrolysis (Hammel et al. 2002). The following enzymatic hydrolysis yield could reach up to 95%. Yu et al. (2009) combined oxidative pretreatment followed by biological treatment with *Pleurotus ostreatus*. At optimum conditions of hydrogen peroxide pretreatment i.e., 2% H_2_O_2_ for 48 h followed by biological pretreatment for 18 days yielded 39.8% total sugar and 49.6% glucose. This was about 5.8 and 6.5 times more as compared to fungal pretreatment alone for 18 days. Saha and Cotta (2007) has reported that peroxide pretreatment under alkaline conditions (addition of NaOH) increased the production of reducing sugars with more than 96% cellulosic conversion as compared to absence of alkali. Cao et al. (2012) performed pretreatment of sweet sorghum bagasse through different pretreatment processes and found the highest yield with dilute NaOH followed by H_2_O_2_ pretreatment. The highest cellulose hydrolysis yield was 74.3%, total sugar yield was 90.9% and ethanol concentration was 0.61% which was 5.9, 9.5, and 19.1 times higher as compared to control.

### Biological pretreatment

In comparison to conventional chemical and physical pretreatment methods, biological pretreatment is considered as an efficient, environmentally safe and low-energy process. Nature has abundant cellulolytic and hemicellulolytic microbes which can be specifically targeted for effective biomass pretreatment (Vats et al. 2013). Biological pretreatments are carried out by microorganisms such as brown, white, and soft-rot fungi which mainly degrade lignin and hemicellulose and little amount of cellulose (Sánchez 2009). Degradation of lignin by white-rot fungi occurs due to the presence of peroxidases and laccases (lignin degrading enzymes) (Kumar et al. 2009). The white-rot fungi species commonly employed for pretreatment are *Phanerochaete chrysosporium, Ceriporia lacerata, Cyathus stercolerus, Ceriporiopsis subvermispora, Pycnoporus cinnarbarinus* and *Pleurotus ostreaus.* Besides these other basidiomycetes species were also studied for breakdown of several lignocellulosic feedstocks. Among these *Bjerkandera adusta, Fomes fomentarius, Ganoderma resinaceum, Irpex lacteus, Phanerochaete chrysosporium, Trametes versicolor*, and *Lepista nuda* are well studied. These species have been reported to show high delignification efficiency (Kumar et al. 2009; Shi et al. 2008). Table 3 summarizes different microorganisms involved in pretreatment strategies and their effects on various biomasses. Pretreatment of wheat straw by fungi (fungal isolate RCK-1) for 10 days resulted in increase of fermentable sugars and decrease in fermentation inhibitors. Although the biological pretreatment is highly intriguing, the rate of hydrolysis of lignocellulosic fractions is too slow which severely hampers to be foreseen as a potential pretreatment method at an industrial scale (Sun and Cheng 2002). In order to make biological pretreatment at par with other pretreatment methods, more basidiomycetes fungi should be tested for its ability to delignify the biomass effectively at a faster rate.

**Table 3:** Different biological pretreatment strategies involved for pretreatment of lignocellulosic biomass and its advantages (adapted from Sindhu et al. 2016)

Microorganism	Biomass	Major effects	References
*Punctualaria* sp. TUFC 20056	Bamboo culms	50% lignin removal	Suhara et al. (2012)
*Irpex lacteus*	Corn stalks	82% of hydrolysis yield	Du et al. (2011)
*Fungal consortium*	Straw	20-fold increase in hydrolysis	Taha et al. (2015)
*P.ostreatus/P.pulmonarius*	Eucalyptus grandis saw dust	20-fold increase in hydrolysis	Castoldi et al. (2014)
*P.chrysosporium*	Rice husk	–	Potumarthi et al. (2013)
Fungal consortium	Corn stover	43.8% lignin removal/sevenfold increase in hydrolysis	Song et al. (2013)
*Ceriporiopsis subvermispora*	Wheat straw	Minimal cellulose loss	Cianchetta et al. (2014)
*Ceriporiopsis subvermispora*	Corn stover	2- to 3-fold increase in reducing sugar yield	Wan and Li (2011)
Fungal consortium	Plant biomass	Complete elimination of use of hazardous chemicals	Dhiman et al. (2015)

#### Combined biological pretreatment

Studies have found that a combination of another pretreatment process with biological pretreatment process is more effective as compared to a single pretreatment process. Wang et al. (2012) combined biological pretreatment with liquid hot water pretreatment method for better enzymatic saccharification of *Populus tormentosa*. This combination reported highest hemicellulose removal (92.33%) resulting in 2.66-fold increase in glucose yield as compared to pretreatment carried out with liquid hot water alone. Yu et al. (2009) studied the novel combination of either physical or chemical pretreatment with biological pretreatment on rice husk. Physical pretreatment was carried out using ultrasound while chemical pretreatment was carried out using H_2_O_2_. Biological pretreatment was carried out using *P. ostreatus*. The combined pretreatment of rice husk carried out using 2% H_2_O_2_ for 48 h along with *P. ostreatus* was found more effective as compared to single step pretreatment using *P. ostreatus* for 60 days. Lignin removal was also found significantly higher as compared to one step treatment. Balan et al. (2008) studied and found that pretreatment of rice husk with *P. ostreatus* followed by AFEX pretreatment produced high glucan and xylan conversion as compared to a single pretreatment with AFEX. The combination of mild acid pretreatment (0.25% H_2_SO_4_) and biological pretreatment using *Echinodontium.taxodii* on water hyacinth was found more effective than one step pretreatment. The reducing sugars yield doubled as compared to single step acid pretreatment method (Ma et al. 2010). Sawada et al. (1995) combined steam explosion and pretreatment by *P. chrysosporium* for the enzymatic saccharification of plant wood. The saccharification of wood increased when treated with *P. chrysosporium* prior to steam explosion. Maximum production of reducing sugar was observed when wood was treated with *P. chrysosporium* for 28 days followed by steam explosion at 215 °C for 60–65 min.

## Applications of biomass pretreatment

Biomass pretreatment results in production of several value-added products. Although, here we have described in brief but this topic is beyond the scope of this review and readers are suggested to refer recent review on various products obtained from pretreated lignocellulosic biomass (Putro et al. 2016). Several valuable products can be obtained through lignocellulosic biomass. Among which biofuel and chemicals are well known and widely studied.

### Biofuels

Several biofuels are obtained through lignocellulosic biomass such as bio-oil, bioethanol, biohydrogen, biogas, syngas etc. Bio-oil is produced through pyrolysis along with biochar, tar and gases. Bio-oil is produced by fast depolymerisation of lignocellulose components viz. hydroxyaldehydes, sugars, hydroxyketones, carboxylic acids, and phenols. Bioethanol can be produced through 5 different methods: separate hydrolysis and fermentation (SHF), simultaneous saccharification and fermentation (SSF), simultaneous and saccharification co-fermentation (SSCF), consolidated bioprocessing (CBP), and integrated bioprocessing (IBP) (Sarkar et al. 2012; Jagmann and Philipp 2014). SSF is the most promising among these processes because of its low-cost and high product yield. IBP is another promising process which involves treatment with microorganisms at every step in a single step. However, there is no reported work on pretreatment through IBP (Chandel et al. 2015). Biohydrogen can be produced from lignocellulosic biomass through thermochemical (gasification and pyrolysis) or biological routes (Ni et al. 2006). Through pyrolysis, hydrogen can be produced through fast or flash pyrolysis (Putro et al. 2016). Hydrogen can produced through gasification by partial oxidation and steam reformation followed by waster-gas shift reaction. Two processes to produce biohydrogen through biological route are: photo fermentation which is light dependant and dark fermentation which is light independent (Sivagurunathan et al. 2016). Although biogas and syngas have similar composition (CO_2_, CH_4_, H_2_, and N_2_), they are produced through two different processes. Biogas is produced through anaerobic digestion which comprises of four steps: hydrolysis, acidogenesis, acetogenesis, and methanogenesis (Taherzadeh and Karimi 2008) while syngas is produced by gasification carried out at lower temperature due to high reactivity of biomass. Biomass gasification has three types of processes namely: (1) pyrolysis which involves anaerobic decomposition of biomass at high temperature, (2) partial oxidation which requires less amount of oxygen as compared to oxidation, and (3) steam gasification which involves the reaction of water with biomass.

### Bioproducts

The chemicals from lignocellulosic biomass can be derived either through carbohydrate source or through lignin. (1) The simplest chemical derived from carbohydrate is furfural and 5-hydroxymethylfurfural (HMF), produced through acid catalyzed dehydration of C_5_ and C_6_ sugars (Delidovich et al. 2016). Sugar alcohols such as sorbitol and xylitol are obtained by the hydrogenation of hexose and pentose (Romero et al. 2016). Also, glycerol, widely used for making bio-solvents, polymers, surfactants etc. can be produced by hydrogenolysis of sorbitol and xylitol (Choi et al. 2015). Also lactic acid and succinic acid can be obtained by the biological conversion caused by bacteria and mold. (2) Lignin has been used to generate heat in the earlier days. In the recent times lignin has been a rich source of valuable products like phenolic compounds. The basic principle behind the conversion of lignin to phenolic compounds is depolymerization. Different ways to convert lignin to phenolics compounds are liquefaction (Kang et al. 2013), oxidation (Ma et al. 2015), solvolysis (Kleinert and Barth 2008), hydrocracking (Yoshikawa et al. 2013) and hydrolysis (Roberts et al. 2011). Lignocellulose biomass has also been used for development of advanced technology products for energy storage, transportation, medical applications, biosensing, environmental remediation etc. (Wang et al. 2013; Brinchi et al. 2013; Yang et al. 2013).

## Conclusion

The presence of lignin in the biomass inhibits the hydrolysis of cellulose and hemicellulose. Therefore, extensive research has been carried out for developing various pretreatment techniques for delignification of biomass. However, critical analysis of pretreatment methods bring us to a conclusion that pretreatment method is a ‘tailor-made’ process for every individual biomass which should be meticulously selected and planned based on the characteristic properties of biomass. Also, it can be concluded that till date a single pretreatment method has not been established which can carry out complete delignification of biomass in an economic and environment friendly manner. Though, combined pretreatment methods have been successful to an extent, still a lot of research needs to be done in developing combined pretreatment methods to their full potential. This critical review comprising of physical, chemical, physicochemical and biological pretreatment processes along with their advantages and disadvantages will help the researcher in planning, selection, and development of pretreatment process for various lignocellulosic biomass.
